# Computational prediction and *in vitro* analysis of the potential ligand binding site within the extracellular ATP receptor, P2K2

**DOI:** 10.1080/15592324.2023.2173146

**Published:** 2023-02-01

**Authors:** Sung-Hwan Cho, Cuong the Nguyen, an Quoc Pham, Gary Stacey

**Affiliations:** a Divisions of Plant Science and Biochemistry, Christopher S. Bond Life Sciences Center, University of Missouri, Columbia, MO, USA; b Plant Genomics and Breeding Institute, Seoul National University, Seoul, Republic of Korea; c Center for Applied Biotechnology and Agricultural High-Tech, Cuu Long Delta Rice Research Institute, Can Tho, Vietnam; d Faculty of Biology and Biotechnology, VNUHCM-University of Sciences, Ho Chi Minh City, Vietnam

**Keywords:** Extracellular ATP, P2K2, purinoreceptor, lectin receptor kinase, *in silico* model

## Abstract

The plant extracellular ATP (eATP) receptor, P2K2, binds eATP with strong ligand affinity through its extracellular lectin domain. Ligand binding activates the intracellular kinase domain of P2K2 resulting in a variety of intracellular responses and, ultimately, increased plant immunity to invading fungal and bacterial pathogens. Here, using a computational prediction approach, we developed a tertiary structure model of the P2K2 extracellular lectin domain. *In silico* target docking of ATP to the P2K2-binding site predicted interaction with several residues through hydrophobic interactions and hydrogen bonding. Our confirmation of the modeling was obtained by showing that H99, R144, and S256 are key residues essential for *in vitro* binding of ATP by P2K2.

## Text

Adenosine 5’-triphosphate (ATP) is widely known for its roles in biosynthetic reactions, intracellular signaling, and energy metabolism, but is also a crucial extracellular signaling molecule. In mammals, extracellular ATP (eATP) is recognized by P2-type purinergic receptors: ionotropic P2X receptors and G-protein-coupled P2Y receptors.^1^ Given the importance of purinergic receptors in cellular processes such as muscle contraction, taste signal transduction, and macrophage activation,^1–3^ studies of the ATP-binding sites of P2Xs and P2Ys have provided important information for developing potential drugs targeting these receptors.^4,5^

In contrast to our understanding of purinergic signaling in animals, much less is known about the role of eATP in plants. However, several papers have implicated eATP in a variety of plant physiological roles, including cell viability,^6^ pollen growth,^7^ root hair development,^8^ thigmotropism,^9^ and gravitropism.^10^ In plants, instead of canonical P2X and P2Y receptors, *Arabidopsis* recognizes eATP through plasma membrane-localized P2K receptors that encode L-type lectin receptor kinases (LecRK).^11,12^ The first plant eATP receptor, P2K1 (LecRK-I.9), regulates ROS production by phosphorylation of RBOHD that controls the stomatal aperture and controls plant defense against *Pseudomonas syringae* and *Phytophthora* species.^13,14^ P2K1 is also involved in plant immune responses through S-acylation.^15^ It was reported that eATP signaling via P2K1 induces plant defense by activating jasmonate signaling.^16^ In addition, P2K1 directly controls defense metabolites via activation of mevalonate kinase in the mevalonate pathway.^17^ P2K1 was also shown to interact and phosphorylate a second eATP receptor P2K2 and contribute to plant defense against pathogens.^12^ A recent study showed that the ROS wave induced by eATP during wounding requires P2K receptors.^18^ Most recently, *CYCLIC NUCLEOTIDE GATED ION CHANNEL2* (*CNGC2*) and *CNGC6* were reported to be involved in eATP-regulated calcium influx.^19,20^

The *Arabidopsis LecRK* gene family is represented by 45 members. It was proposed that gene transposition duplication and tandem duplication events gave rise to this diverse *LecRK* family.^21,22^ Various members of this LecRK family have been shown to play crucial roles in plants. For example, LecRK-I.1 functions in the regulation of cell death following insect egg recognition.^23^ LecRK-I.8 was shown to be an extracellular nicotinamide adenine dinucleotide (NAD^+^) receptor and was also involved in early steps of egg perception.^23,24^ LecRK-IX.1 and LecRK-IX.2 regulate *Phytophthora* resistance and plant cell death.^25^ In addition, LecRK-IX.2 is required for the activation of flg22-induced pathogen associated molecular pattern (PAMP) triggered immunity (PTI).^26^ LecRK-VI.2 promotes plant defense against both biotrophic and necrotrophic pathogens as a positive regulator of PTI.^27^ LecRK-V.2 and LecRK-VII.1 are involved in JA-mediated stomatal immunity.^28^

The P2K purinoreceptors consist of an extracellular legume-lectin-like domain that can bind ATP with high affinity, a single transmembrane domain, and an intracellular serine/threonine kinase domain.^11,12,29^ The first plant purinergic receptor to be isolated, P2K1 from *Arabidopsis thaliana*, exhibited high ATP binding affinity (*K*_d_ = 45.7 ± 3.1 nM, *B*_max_ = 488.0 ± 6.3 pmol/mg).^11^ Subsequently, a second purinergic receptor P2K2 (LecRK-I.5) was identified by complementation of the *p2k1* mutant phenotype.^12^ P2K2 belongs to the LecRK clade I, which also includes P2K1. Interestingly, P2K2 binds to ATP with slightly higher binding affinity (*K*_d_ = 44.5 ± 15.73 nM, *B*_max_ = 625.8 ± 86.48 pmol/mg) than P2K1.^12^

In a previous study, we performed an *in silico* study of the molecular interaction between P2K1 and ATP with the reported crystal structure data sets from 54 L-type lectin proteins.^30,31^ The blind docking analysis demonstrated that ATP binds to four loops (loops A, B, C, and D) of the P2K1 lectin domain with high affinity.^30^ The computational target docking model of ATP to the P2K1-binding site predicted interaction with 12 residues on the four loops.^30^ The model was validated by *in vitro* ATP-binding assays with the purified extracellular lectin domain of wild-type and various mutant forms of P2K1.^30^ This study implicated two loops (loops B and C) within the P2K1 structure as being critical for ATP binding.^30^ However, specific details about the location and composition of the ATP binding site remain to be elucidated.

**Figure 1.**
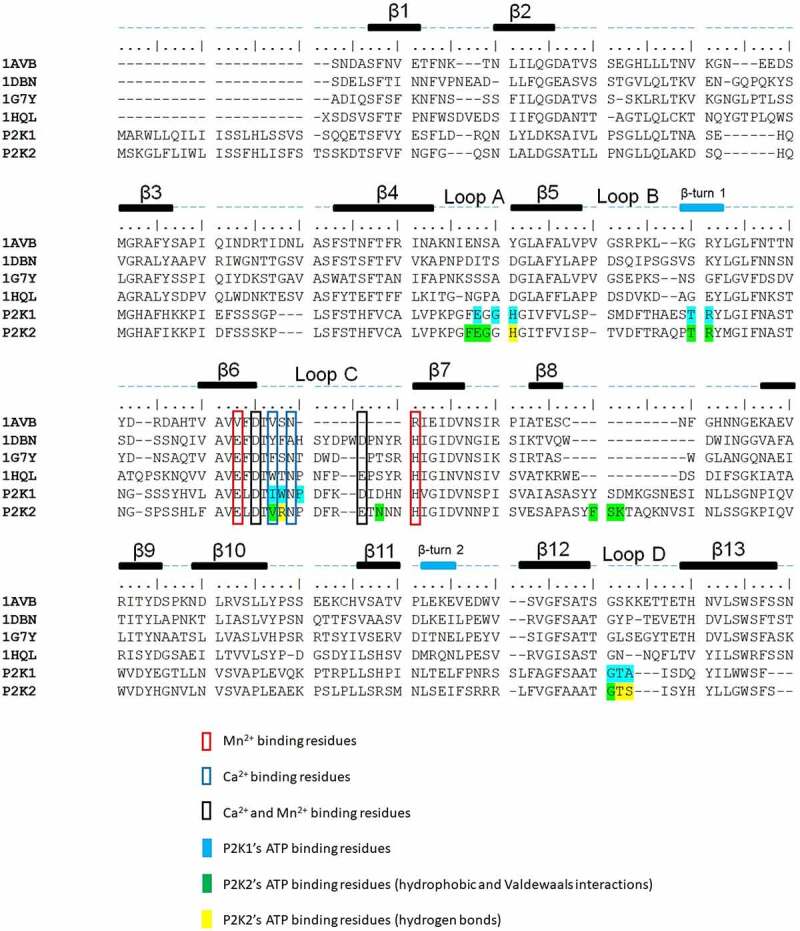
Multiple sequence alignments of P2K2 and P2K1 extracellular domains by comparing four crystalized lectin proteins. 1AVB (*Phaseolus vulgaris*), 1DBN (*Maackia amurensis*), 1G7Y (*Dolichos biflorus*) and 1HQL (*Griffonia simplicifolia*). The figure was generated using Bioedit.

In order to define the putative ATP-binding site within the P2K2 lectin domain, we used computational approaches to predict the protein structure of P2K2 and to dock eATP to the protein to identify the key amino acids involved in binding. Among 40 P2K2 homologous crystal structures present in the Protein Data Bank (www.rcsb.org), P2K1 and four candidates with the highest sequence identity to P2K2 were chosen: 1G7Y (lectin from the legume *Dolichos biflorus*, 26%), 1DBN (lectin from the legume tree *Maackia amurensis*, 26.3%), 1HQL (lectin from the legume *Griffonia simplicifolia*, 26.7%), and 1AVB (lectin from common bean *Phaseolus vulgaris*, 25.1%) (Figure 1). P2K1 and P2K2 share 9, predicted ATP-binding residues including E96, H99, T117, R118, V143 (I143 in P2K1), R144 (W144 in P2K1), G244 (G245 in P2K1), T245 (T246 in P2K1), and S246 (A247 in P2K1) over 15 and 11 putative ATP-binding residues of P2K2 and P2K1, respectively (Figure 1). The larger number of ATP-binding residues in P2K2 may reflect the higher binding affinity for ATP than P2K1.

**Figure 2.**
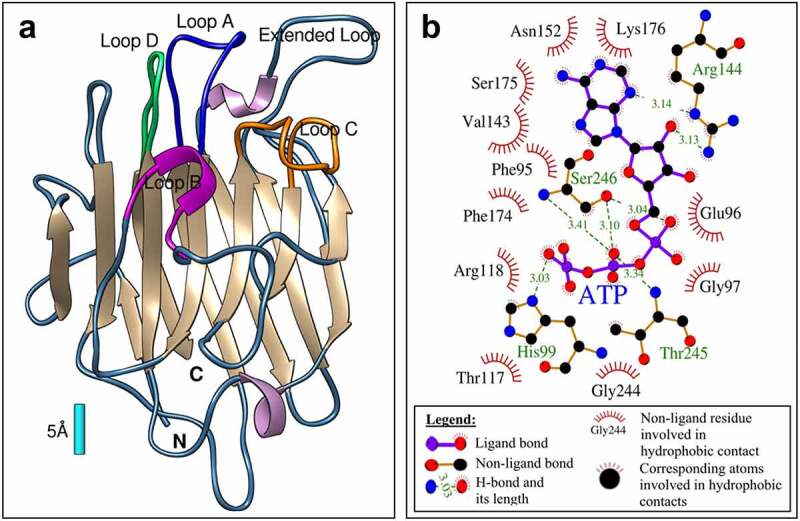
Computational prediction of the ATP binding within the P2K2 extracellular domain (ectodomain). (a) P2K2 ectodomain 3D structure was predicted using homology modeling. The putative backbone alpha-carbon trace of P2K2 includes 13 β-strands (brown); 4 defined loops (A-blue; B-magenta; C-Orange; D-green); an extended loop (light blue) and 2 β-turns (purple). The bar shows relative scale (1 angstrom = 10^−10^ m). (b) A detailed map of interaction between P2K2 and ATP in the docking complex was predicted using target docking. ATP resides in the middle of the map and is surrounded by interacting residues, green dashed-lines with green numbers are hydrogen bonds with bond distances in angstroms shared with atoms of ATP, where black residues with a red crown denote hydrophobic and Van der Waals interactions. Numbered residues represent their actual position in the P2K2 full-length sequence.

Starting from these structures, Modeler v9.21 (https://salilab.org/modeler/) was used to predict the P2K2 extracellular domain structure. The quality of the raw P2K2 modeling structure was checked with Ramachandran plots (https://bip.weizmann.ac.il/toolbox/structure/validation.htm). Finally, the refined P2K2 structure model was visualized. As expected, the putative extracellular domain structure of P2K2 is very similar to that of P2K1, containing 13 β-strands, four defined loops (named loop A, B, C, and D), two β-turns, and an extended loop (Figure 2a). Sequence-based and structural-based methods were used to predict the ATP binding site within P2K2. Using four prediction tools (COACH, COFACTOR, FindSite, and TM-Site), we obtained similar binding site prediction (Table 1).

**Table 1.** List of predicted P2K2 interacting residues with ATP ligand obtained from different prediction tools.

Prediction tools	LOOP A	LOOP B	LOOP C	LOOP D	Score*
COACH	Gly98, His99	Gln115, Thr117, Arg118	Val143, Asn145	Gly244, Thr245, Ser246	0.56
COFACTOR	Gly98, His99	Thr117, Arg118	Val143, Asn145	Gly244, Thr245, Ser246	0.52
FindSite	Gly98, His99	Thr117, Arg118	Val143, Asn145	Gly244, Thr245, Ser246	0.55
TM-Site	Gly98, His99	Thr117, Arg118	Val143, Asn145	Gly244, Thr245, Ser246	0.40
Consensus residues	Gly98, His99	Thr117, Arg118	Val143, Asn145	Gly244, Thr245, Ser246	

* Values range in between [0–1]; where a higher score indicates a more reliable ligand-binding site prediction.

**Figure 3.**
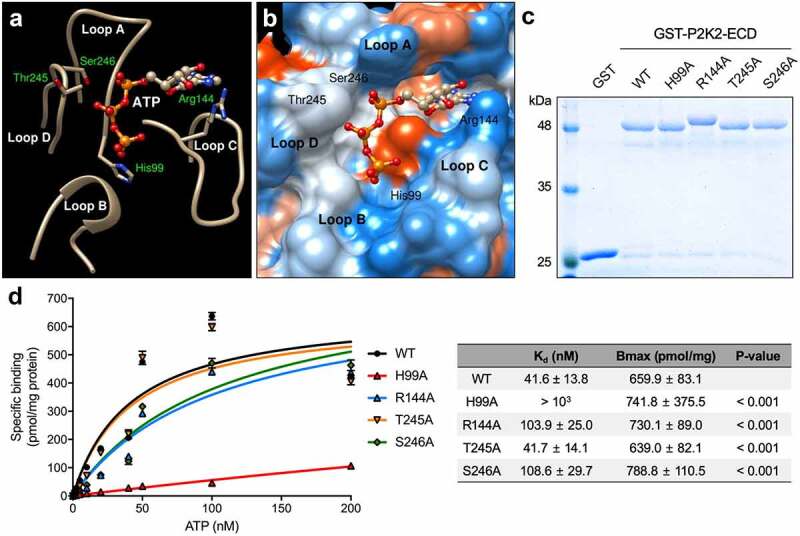
Cartoon representations of the predicted ATP-binding site and interaction map between ATP and residues of P2K2. P2K2 binds ATP *in vitro*. (a) A close-up of the P2K2-binding loops and ATP molecule in ball and stick mode. (b) A close up of the electrostatic potential molecular surface of the P2K2-binding pocket and ATP molecule in ball and stick mode. (c) SDS-PAGE gel showing GST-tag purified proteins expressed in *E. coli* cells expressing GST-tagged P2K2-extracellular domain WT, H99A, R144A, T254A, and S246A. GST proteins were used as control. The protein was measured by Coomassie Brilliant Blue staining. (d) *In vitro* binding of ^32^P-labeled ATP to ectodomain of P2K2 wild-type and mutant versions. After incubating P2K2 wild-type or mutant versions with indicated concentrations of ^32^P-labeled ATP (PerkinElmer, specific activity 800 Ci mmol^−1^) in a 100 µl reaction buffer including 10 mM HEPES (pH 7.5), 5 mM MgCl_2_. The bound ATP and free ATP were separated by Sephadex PD MiniTrap G-25 gel filtration columns (GE Healthcare). The bound radio ligand collected in scintillation vials. After mixing with scintillation cocktail (MP Biomedicals), the signal of bound radio ligand was measured using liquid scintillation counting (Tri-Carb 2810TR, PerkinElmer). Data were plotted as a specific binding with SEs of three replications. The dissociation constant (K_d_) and maximum binding capacity (B_max_) were calculated by a non-linear regression model analysis using GraphPad Prism 7. P-value shows statistical difference of mutant versions to the wild-type.

The putative ATP binding site is located between four defined loops with nine consensus-binding residues (Figure 2 and Table 1). This predicted binding site was used to predict ATP-P2K2 target docking using AutoDockVina (http://vina.scripps.edu/). In the ATP-P2K2 target docking model, ATP is predicted to bind to 15 residues located within loops A, B, C, D, and the extended loop (Figure 2b and Table 2). Based on all analyses, the best putative ATP-P2K2 binding complex model showed the lowest energy score (−8.1 kcal/mol) within 4 predicted residues (Figure 3a and 3B).

**Table 2.** List of P2K2 interacting residues with the ATP ligand obtained from target docking.

Loop	Hydrogen bonds	Hydrophobic and Val de Waals interactions
A	His99	Phe95, Glu96, Gly97
B	-	Thr117, Arg118
C	Arg144	Val143, Asn152, Phe174, Ser175, Lys176
D	Thr245, Ser246	Gly244
**Total**	**4**	**11**

In order to determine whether the four predicted residues [histidine 99 (H99), arginine 144 (R144), threonine 245 (T245), and serine 246 (S246)] are critical for ATP binding, we performed site-directed mutagenesis to product alanine substitutions for each of these four residues, specifically H99A, R144A, T245A, and S246A. The wild-type and mutated constructs were transformed into *E. coli* BL21-AI. We then purified the GST-fused extracellular domain proteins and conducted *in vitro* binding assays using radiolabeled γ^32^P-ATP. We previously showed that P2K2 had a slightly higher ATP binding affinity than P2K1.^12^ Similar to the previous result (*K*_d_ = 44.5 ± 15.73 nM, *B*_max_ = 625.8 ± 86.48 pmol/mg),^12^ the wild-type P2K2 protein showed a typical saturation curve for ATP binding with high affinity (*K*_d_ = 41.6 ± 13.8 nM, *B*_max_ = 659.9 ± 83.1 pmol/mg) (Figure 3d).

Of those key amino acid residues predicted to interact with ATP in the P2K2 structure, mutation of the H99 residue resulted in a significant reduction in ATP binding ability (Figure 3d). Interestingly, the region of H99 is structurally close to the predicted region for binding of the gamma-phosphate of ATP, suggesting that H99 may be a critical residue for ATP-P2K2 binding. Many carbohydrate-binding lectin proteins have a conserved aspartic acid at this site, instead of H99 in P2K1 and P2K2, perhaps explaining why these P2K proteins recognize nucleotides and not sugars.^32–34^ Consistent with such a possibility, LecRK-I.8 with N99, instead of H99, was shown to bind to NAD^+^, and not ATP.^24^ Mutation in R144 and S246 resulted in a significant reduction in the binding activity, which further confirms our predicted P2K2 structure (Figure 3d). The S246 residue is predicted to interact with the beta-phosphate of ATP, which can be essential for the binding of ADP. Interestingly, the amino acid 247 at P2K1, which is located with S246 of P2K2, has an alanine (Figure 1). Previously, the deletion from G245 to A247 in P2K1 showed no effect on ATP binding affinity compared to the wild-type.^30^ Therefore, it is possible that the higher ATP binding affinity of P2K2 than P2K1 may be affected by S246 residue. However, mutation in T245 showed a similar ATP binding affinity compared to the wild-type P2K2 (Figure 3d).

Mammals have several P2 receptors; for example, seven P2X and eight P2Y receptors have been cloned from mammals.^35^ Hence, it is quite possible that plants possess other receptors, in addition to P2K1 and P2K2, better knowledge of the requirements for ATP binding by plant receptors will be helpful in the identification of possible, novel plant receptors. In addition, a forward-genetic screen strategy with an eATP-antagonist, such as Suramin and PPRAD, may be helpful to isolate new P2K receptors.
